# Evening chronotype is associated with higher circulating pyruvate levels in adults

**DOI:** 10.3389/fnut.2026.1839777

**Published:** 2026-07-23

**Authors:** Sarang Jeong, Eunjin Jang, Jinhyun Kim, Sang Youl Rhee, Sohyun Park

**Affiliations:** 1 Department of Food and Nutrition, Industry-Academy Collaboration Foundation, Dongduk Women’s University, Seoul, Republic of Korea; 2 The Korean Institute of Nutrition, Hallym University, Chuncheon, Republic of Korea; 3 Department of Food Science and Nutrition, Hallym University, Chuncheon, Republic of Korea; 4 Department of Endocrinology and Metabolism, Center for Digital Health, Kyung Hee University College of Medicine, Seoul, Republic of Korea

**Keywords:** chronotype, citric acid cycle, metabolomics, nuclear magnetic resonance, pyruvates

## Abstract

**Background/objectives:**

Chronotype reflects interindividual differences in the circadian timing system. Animal studies suggest reduced mitochondrial oxidative metabolism and tricarboxylic acid (TCA) cycle activity in individuals with an evening chronotype; however, human evidence linking chronotype to mitochondrial metabolic alterations remains limited. This cross-sectional study examined whether chronotype is associated with differences in circulating TCA-related metabolites, including pyruvate, lactate, and citrate.

**Methods:**

A total of 272 adults from the 2022 Gangwon Obesity and Metabolic Syndrome Cohort were classified as having morning (≥18), intermediate (12–17), or evening (≤11) chronotype using the reduced Morningness–Eveningness Questionnaire. Fasting serum concentrations of pyruvate, lactate, and citrate were measured. Associations were examined using general linear models adjusted for fasting status; demographic, socioeconomic, and lifestyle factors; as well as body mass index. Sensitivity analyses excluded participants taking metabolic disease-related medications.

**Results:**

Major clinical and metabolic indicators did not differ across chronotypes. Serum pyruvate concentrations were higher in the evening chronotype group than in the morning chronotype group across all adjusted models (*p* < 0.048), whereas lactate and citrate concentrations did not differ. These findings remained consistent in sensitivity analyses excluding participants with metabolic diseases (*p* = 0.006–0.010).

**Conclusion:**

Across analyses that included and excluded participants using metabolic disease-related medications, evening chronotype was consistently associated with higher circulating pyruvate concentrations, a key metabolic branch point downstream of glycolysis. These findings suggest chronotype-related differences in pyruvate metabolism, with potential implications for metabolic regulation.

## 1 Introduction

Chronotype reflects interindividual differences in the circadian timing system and manifests as variation in sleep–wake timing, including preferred bedtimes and wake times (1, 2). Circadian rhythm differences influence not only sleep-related hormone secretion (3) but also insulin sensitivity (4), dietary behaviors (5), and glucose regulation (6, 7), thereby affecting overall metabolic homeostasis. In addition, chronotype is associated with various behavioral and lifestyle factors, including adiposity, smoking, alcohol consumption, and socioeconomic status (8), which may contribute to metabolic disease risk and influence metabolic phenotypes. Epidemiological studies have consistently reported differences in the prevalence of metabolic diseases according to chronotype, with the evening chronotype associated with a higher risk of obesity and type 2 diabetes (9, 10). Despite these associations, mechanistic evidence in humans remains limited regarding the biological pathways linking the increased metabolic disease risk observed in individuals with an evening chronotype to impaired glucose regulation and insulin resistance. Elucidating differences in oxidative metabolic processes associated with chronotype may provide important insights into the mechanisms underlying these relationships.

The tricarboxylic acid (TCA) cycle, also known as the citric acid cycle, is a central mitochondrial metabolic pathway in which the final oxidative stages of carbohydrate, fatty acid, and amino acid metabolism converge. It plays a critical role in energy production and the generation of reducing equivalents (11). In this pathway, pyruvate, the end product of glycolysis, is converted to acetyl-CoA by the pyruvate dehydrogenase complex and subsequently enters the TCA cycle. When oxidative metabolism is impaired, pyruvate may accumulate and be converted to lactate (12). Citrate, formed by the condensation of acetyl-CoA and oxaloacetate, is an early TCA cycle intermediate that may reflect substrate entry into the cycle and overall TCA cycle activity (13). Accordingly, pyruvate, lactate, and citrate are metabolites involved in glycolytic and TCA-cycle metabolism. Evaluating their circulating concentrations according to chronotype may therefore provide insight into chronotype-related differences in metabolic regulation, particularly in individuals with an evening chronotype, who are at an increased risk of metabolic disease.

When mitochondrial oxidative metabolism is reduced, pyruvate entry into the TCA cycle may be limited, disrupting the balance between glycolysis and oxidative phosphorylation and promoting pyruvate and lactate accumulation (14). Such alterations may contribute to redox imbalance, impaired insulin signaling, and reduced energy efficiency (15). Furthermore, restricted substrate entry into the TCA cycle may decrease citrate production by limiting the condensation of acetyl-CoA and oxaloacetate, thereby reducing overall TCA cycle flux and efficiency (14, 16). These changes may subsequently affect metabolic signaling pathways involved in fatty acid oxidation and glucose metabolism (16). Consequently, alterations in TCA-related metabolites may reflect early metabolic disturbances that precede overt clinical abnormalities and provide mechanistic insight into the association between chronotype and metabolic disease risk.

A previous mouse study demonstrated that loss of BMAL1, which disrupts circadian rhythmicity, impairs mitochondrial gatekeeper function and promotes abnormal lactate accumulation by shifting metabolism toward glycolysis (17). As core clock genes regulate mitochondrial oxidative phosphorylation, monitoring metabolites such as pyruvate, lactate, and citrate may provide critical insight into how circadian disruption contributes to metabolic imbalance by altering cellular energy homeostasis (18). These findings suggest that coordinated regulation of the biological clock and mitochondrial oxidative processes is essential for maintaining systemic energy homeostasis.

Although the circadian clock is known to regulate the expression of enzymes involved in mitochondrial oxidative metabolism (18), the extent to which these molecular changes translate into measurable metabolic differences among human chronotypes remains poorly understood. Cellular and animal studies have demonstrated that circadian disruption reduces mitochondrial activity (17) and impairs TCA cycle function, yet corresponding evidence in humans remains limited. Furthermore, despite the biological importance of pyruvate, lactate, and citrate as indicators of mitochondrial metabolism, no study has determined whether chronotype, particularly eveningness, is associated with altered concentrations of these metabolites.

Using baseline data from the 2022 Gangwon Obesity and Metabolic Syndrome study, this cross-sectional study aimed to evaluate whether serum concentrations of pyruvate, lactate, and citrate—metabolites that indirectly reflect mitochondrial oxidative metabolism and TCA cycle function—differ according to chronotype. Through this approach, we sought to provide foundational evidence to improve understandings of the metabolic characteristics associated with the evening chronotype while offering exploratory insight into metabolic alterations that may occur during the early stages of metabolic dysfunction.

## 2 Materials and methods

### 2.1 Study population

Data for the present analysis were obtained from the Gangwon Obesity and Metabolic Syndrome study, which included adults recruited from mountainous regions of Gangwon Province between June 18 and July 3, 2022. Among 319 adults aged 20–69 years, 15 participants with missing data on metabolites, chronotype, or blood tests were excluded. To identify implausible dietary reports, an interquartile range (IQR)-based method was applied (19). The 25th (Q1) and 75th (Q3) percentiles of daily energy intake were calculated, and values exceeding 1.5 × IQR (Q3–Q1) were considered outliers. The lower cutoff was set to Q1–1.5 × IQR but constrained to a physiologically plausible minimum of 600 kcal (20), whereas the upper cutoff was set to Q3 + 1.5 × IQR. Participants with energy intake outside this range were excluded (*n* = 32), resulting in a final analytic sample of 272 participants. The study protocol was approved by the Institutional Review Board of Hallym University (HIRB-2021-079-2-RR-CR).

### 2.2 Chronotype assessment using the reduced Morningness–Eveningness Questionnaire

Chronotype was assessed using the reduced Morningness–Eveningness Questionnaire (MEQ-reduced) (21). The questionnaire comprises five items that assess preferred wake time under free conditions, degree of fatigue during the first 30 min after awakening, preferred bedtime based on evening sleepiness, time of day when individuals feel their best, and self-perceived chronotype. Responses were summed to generate a total score. Participants were classified as morning (18–25 points), intermediate (12–17 points), or evening (4–11 points) chronotypes. The internal consistency of the MEQ-reduced was assessed using Cronbach’s alpha.

### 2.3 General characteristics

Covariates included age, sex, education level (≤elementary school, middle school, high school, or ≥college), household income (low, middle, high), and occupation (office work, manual labor, sales/service, or other [military personnel, homemakers, students, unemployed individuals]). Alcohol consumption was categorized as current drinking (≥1 time/wk) or non-drinking (never or former drinking). Smoking status was classified as ever smoking (≥100 cigarettes during the lifetime) or never smoking (<100 cigarettes during the lifetime or no smoking history). Physical activity was assessed using the International Physical Activity Questionnaire and expressed as metabolic equivalent task minutes per week (MET-min/wk) (22). MET values of 3.3, 4.0, and 8.0 were assigned to low-, moderate-, and vigorous-intensity activities, respectively. Total physical activity was calculated as the sum of MET-min/wk. across all activity intensities.

### 2.4 Bedtime, wake time, sleep duration, and social jetlag

Bedtime and wake time on weekdays and weekends were assessed using structured questionnaire items. Sleep duration was calculated separately for weekdays and weekends based on the reported sleep and wake times (23). Social jetlag was defined as the absolute difference between weekday and weekend mid-sleep times. Mid-sleep time was calculated as bedtime plus one-half of the sleep duration (mid-sleep = bedtime + sleep duration/2).

### 2.5 Anthropometric measurements and blood pressure

Anthropometric measurements were obtained after a minimum of 8 h of overnight fasting, with participants wearing light clothing. Height was measured without shoes using a stadiometer, and waist and hip circumferences were measured to the nearest centimeter by trained staff. Body weight, body mass index (kg/m^2^), fat mass (kg), body fat percentage (%), visceral fat area (cm^2^), and visceral fat mass (kg) were assessed using a bioelectrical impedance analyzer (ACCUNIQ BC720; SELVAS Healthcare Inc., Daejeon, Korea). Blood pressure was measured after participants rested in a seated position for 5–10 min using an automated sphygmomanometer (BP500; SELVAS Healthcare).

### 2.6 Dietary intake assessment using a food frequency questionnaire

To minimize the acute effects of recent dietary intake, all blood samples were collected after an 8-h overnight fast (24). Usual dietary intake patterns were assessed using a validated food frequency questionnaire. The results are presented in Supplementary Table 3.

### 2.7 Metabolite and biochemical measurements

Serum concentrations of pyruvate, lactate, and citrate were measured in blood samples collected after a minimum 12-h overnight fast by trained clinical personnel. Blood was collected in serum separator tubes, and serum samples were stored at −80 °C until analysis. Metabolite concentrations were quantified using a standardized nuclear magnetic resonance–based platform under uniform analytical conditions, with quality control performed according to internal standards. Raw metabolite concentrations were used for all statistical analyses. Serum levels of aspartate aminotransferase, alanine aminotransferase, insulin, glucose, triglycerides, total cholesterol, low-density lipoprotein cholesterol, and high-density lipoprotein cholesterol were also measured. Detailed analytical procedures are provided in Supplementary material.

### 2.8 Statistical analysis

All statistical analyses were performed using SAS version 9.4 (SAS Institute Inc., Cary, NC, USA). Differences in categorical variables across chronotypes were analyzed using Fisher’s exact test because of the small number of participants in the evening chronotype group (<30 participants). Results are presented as frequencies and percentages. Differences in continuous variables were assessed using the Wilcoxon rank-sum test with Dwass–Steel–Critchlow–Fligner *post hoc* comparisons to account for unequal group sizes and potential departures from normality. Differences in serum metabolite concentrations across chronotypes were analyzed using general linear models with progressive adjustment for potential confounders. Model 1 was adjusted for age, sex, and fasting status. Model 2 was additionally adjusted for education level and household income. Model 3 was further adjusted for body mass index, alcohol consumption, and smoking status. Fasting status was categorized as non-fasting (<8 h) or fasting (≥8 h) according to the study protocol and was included as a covariate in all adjusted models. Adjusted least-squares means were compared using Tukey–Kramer post-hoc tests. Statistical significance was defined as *p* < 0.05.

## 3 Results

### 3.1 General characteristics according to chronotype assessed by the MEQ-reduced

General characteristics according to chronotype are presented in Table 1. Based on the MEQ-reduced scores, participants were classified as morning (*n* = 132), intermediate (*n* = 120), or evening (*n* = 20) chronotypes. The mean MEQ-reduced scores were 20.2 ± 1.8, 14.6 ± 1.6, and 10.3 ± 0.9 for the morning, intermediate, and evening chronotypes, respectively. The standardized Cronbach’s alpha coefficient for the MEQ-reduced was 0.634, indicating acceptable internal consistency. Participants with an evening chronotype were younger (55.8 ± 7.1 years) than those with a morning chronotype (59.6 ± 6.8 years) or an intermediate chronotype (56.2 ± 7.0 years; *p* < 0.001). The proportion of participants with a college education or higher was greater in the evening chronotype group (25.0%) than in the morning chronotype group (18.2%); however, the difference was not statistically significant. No significant differences were observed across chronotypes in sex, household income, occupation, alcohol consumption, smoking status, or physical activity levels, expressed as MET-min.

**Table 1:** General characteristics of study participants according to chronotype assessed using the MEQ-reduced.

Characteristic	Category	Total (*n* = 272)			*p*-value
		Morning chronotype (*n* = 132)	Intermediate chronotype (*n* = 120)	Evening chronotype (*n* = 20)	
MEQ-reduced scores		20.2 ± 1.8	14.6 ± 1.6	10.3 ± 0.9	-
Age	Years	59.6 ± 6.8^b^	56.2 ± 7.0^a^	55.8 ± 7.1^a^	<0.001
Sex	Male/female	43 (32.6)/89 (67.4)	26 (21.7)/94 (78.3)	5 (25.0)/15 (75.0)	0.139
Education level	Elementary school or less	33 (25.0)	14 (11.7)	3 (15.0)	0.052
	Middle school or less	27 (20.5)	18 (15.0)	5 (25.0)	
	High school	48 (36.4)	53 (44.2)	7 (35.0)	
	College or higher	24 (18.2)	35 (29.2)	5 (25.0)	
Household income	Lowest	42 (32.1)	32 (26.9)	5 (26.3)	0.206
	Middle	54 (41.2)	48 (40.3)	4 (21.1)	
	Highest	35 (26.7)	39 (32.8)	10 (52.6)	
Occupation	Non-manual workers	16 (12.1)	23 (19.2)	3 (15.0)	NA
	Manual workers	42 (31.8)	26 (21.7)	0 (0.0)	
	Service and sales workers	26 (19.7)	29 (24.2)	14 (70.0)	
	Others (e.g., soldiers, homemakers, students, and the unemployed)	48 (36.4)	42 (35.0)	3 (15.0)	
Drinking status^(1)^		81 (61.8)	72 (60.0)	15 (75.0)	0.449
Smoking status^(2)^		29 (22.3)	24 (20.2)	5 (25.0)	0.827
MET-min/wk^(3)^ (*n* = 166)		1,313.5 ± 944.0	1,214.7 ± 975.1	858.8 ± 586.6	0.222

Data are presented as mean ± standard deviation (SD) or weighted frequency (%). The *p*-value for age was obtained using the Kruskal–Wallis test with post hoc multiple comparisons. All other *p*-values were obtained using Fisher’s exact test.

(1) Drinking status was defined as alcohol consumption at least once per week.

(2) Smoking status was defined as having smoked at least five packs of cigarettes during the lifetime.

(3) MET-min/wk = metabolic equivalent task minutes per week.

Different superscript letters (a–c) indicate significant differences between groups based on post-hoc pairwise comparisons.

### 3.2 Weekday and weekend bedtime, wake time, sleep duration, and social jetlag according to chronotype

Weekday and weekend bedtimes, wake times, sleep durations, and social jetlag by chronotype are presented in Table 2. Bedtime and wake time on both weekdays and weekends differed significantly across chronotypes (all *p* < 0.001). On weekdays, the average bedtime was 22:10 in the morning chronotype and 00:26 in the evening chronotype, whereas the corresponding wake times were 05:20 and 06:11, respectively. On weekends, the average bedtime was 22:24 in the morning chronotype and 00:36 in the evening chronotype, with corresponding wake times of 05:54 and 06:57, respectively. Weekday and weekend sleep durations did not differ significantly across chronotypes. Weekday sleep duration ranged from 6.8 to 7.3 h, whereas weekend sleep duration ranged from 7.8 to 8.8 h. Social jetlag increased progressively from the morning chronotype (31.7 min) to the intermediate chronotype (44.5 min) and evening chronotype (59.8 min); however, the difference did not reach statistical significance (*p* = 0.052).

**Table 2:** Bedtime, wake time, sleep duration, and social jetlag according to chronotype.

Sleep variables	Total (*n* = 272)			*p*-value
	Morning chronotype (*n* = 132)	Intermediate chronotype (*n* = 120)	Evening chronotype (*n* = 20)	
Weekday sleep				
Bedtime (hh:mm)	22:10 (01:03)^b^	23:17 (01:22)^b^	00:26 (01:54)^a^	<0.001
Wake-up time (hh:mm)	05:20 (00:57)^a^	6:10 (02:00)^b^	6:11 (04:06)^c^	<0.001
Sleep duration (h)	7.2 (0.2)	7.3 (0.2)	6.8 (0.4)	0.331
Weekend sleep				
Bedtime (hh:mm)	22:24 (01:28)^b^	23:42 (01:59)^b^	00:36 (03:51)^a^	<0.001
Wake-up time (hh:mm)	05:54 (01:16)^a^	07:00 (01:24)^b^	06:57 (4:38)^c^	<0.001
Sleep duration (h)	7.8 (0.2)	7.8 (0.3)	8.8 (0.6)	0.218
Social jetlag (min)	31.7 (5.6)	44.5 (6.0)	59.8 (13.3)	0.052

Data are presented as mean (SD). *p*-values were derived from general linear models for age, sex, fasting status, education level, household income, alcohol consumption, and smoking status. Sleep duration was calculated in minutes and converted to hours for presentation. Different superscript letters (a–c) indicate significant differences between groups based on post-hoc pairwise comparisons.

### 3.3 Adjusted least-squares means of serum pyruvate, lactate, and citrate across hierarchical models according to chronotype

Table 3 presents adjusted least-squares means for serum pyruvate, lactate, and citrate concentrations according to chronotype across hierarchical models. In the overall population, serum pyruvate concentrations increased progressively from the morning to the evening chronotype. The overall effect of chronotype remained significant across all models (Model 1: *p* = 0.038; Model 2: *p* = 0.043; Model 3: *p* = 0.045), and pairwise comparisons showed significantly higher pyruvate concentrations in the evening chronotype than in the morning chronotype in all models (all *p* < 0.05). Lactate concentrations showed a similar pattern of increase. Pairwise comparisons indicated significantly higher lactate concentrations in the evening than in the morning chronotype across all models (Model 1: *p* = 0.043; Model 2: *p* = 0.027; Model 3: *p* = 0.033), although the overall effect of chronotype reached significance only in Model 2 (*p* = 0.046). Citrate concentrations tended to decrease from the morning to the evening chronotype. Although the overall effect of chronotype was not significant in any model (Model 1: *p* = 0.059; Model 2: *p* = 0.096; Model 3: *p* = 0.110), pairwise comparisons showed significantly lower citrate concentrations in the evening chronotype than in the morning chronotype in Models 1 (*p* = 0.021) and 2 (*p* = 0.040). In Model 3, the difference showed a marginal trend toward significance (*p* = 0.051). Among participants not using metabolic disease-related medication, associations between chronotype and metabolite concentrations were generally stronger. Pyruvate concentrations were significantly higher in the evening chronotype across all models (overall *p* = 0.007–0.010; pairwise *p* = 0.002–0.005). Lactate concentrations were consistently higher in the evening chronotype than in the morning chronotype across all models, with significant overall differences observed in Models 1 and 2. Citrate concentrations were consistently lower in the evening chronotype than in the morning chronotype, with significant overall and pairwise differences observed in Model 1. The pyruvate/lactate ratio did not differ significantly according to chronotype in either the primary or sensitivity analyses (Supplementary Table 5).

**Table 3:** Adjusted associations of chronotype with serum pyruvate, lactate, and citrate concentrations in all participants and after excluding participants using metabolic disease-related medications.

Metabolite	Chronotypes	*n*	Model 1	Model 2	Model 3
All participants (*n* = 272)					
Pyruvate	Morning	132	0.072 (0.053–0.091)	0.069 (0.050–0.089)	0.069 (0.051–0.089)
	Intermediate	120	0.074 (0.055–0.093)	0.072 (0.053–0.091)	0.071 (0.052–0.090)
	Evening	20	0.084 (0.063–0.104)	0.081 (0.060–0.102)	0.081 (0.060–0.102)
	Overall *p*^(1)^		0.038	0.043	0.045
	Pairwise *p*^(2)^ (evening vs. morning)		0.011	0.013	0.013
	Pairwise *p*^(2)^ (intermediate vs. morning)		0.349	0.332	0.553
	Adjusted *R*^2^^(3)^		0.042	0.048	0.116
Lactate	Morning	132	1.742 (0.982–2.503)	1.744 (0.979–2.509)	1.696 (0.924–2.468)
	Intermediate	120	1.894 (1.140–2.648)	1.913 (1.156–2.670)	1.828 (1.064–2.593)
	Evening	20	2.114 (1.290–2.938)	2.168 (1.329–3.000)	2.100 (1.261–2.938)
	Overall *p*^(1)^		0.077	0.046	0.076
	Pairwise *p*^(2)^ (evening vs. morning)		0.043	0.027	0.033
	Pairwise *p*^(2)^ (intermediate vs. morning)		0.125	0.091	0.184
	Adjusted *R*^2^^(3)^		0.017	0.020	0.039
Citrate	Morning	129	0.066 (0.056–0.076)	0.066 (0.056–0.076)	0.064 (0.054–0.074)
	Intermediate	118	0.064 (0.054–0.074)	0.064 (0.054–0.074)	0.063 (0.053–0.073)
	Evening	19	0.060 (0.049–0.071)	0.061 (0.050–0.072)	0.060 (0.048–0.074)
	Overall *p*^(1)^		0.059	0.096	0.110
	Pairwise *p*^(2)^ (evening vs. morning)		0.021	0.040	0.051
	Pairwise *p*^(2)^ (intermediate vs. morning)		0.238	0.219	0.196
	Adjusted *R*^2^^(3)^		0.076	0.068	0.095
Participants without metabolic disease-related medication^(4)^ use (*n* = 127)					
Pyruvate	Morning	55	0.050 (0.045–0.055)	0.049 (0.044–0.055)	0.052 (0.046–0.058)
	Intermediate	61	0.051 (0.046–0.057)	0.050 (0.044–0.057)	0.051 (0.045–0.058)
	Evening	11	0.068 (0.057–0.078)	0.068 (0.057–0.079)	0.068 (0.057–0.079)
	Overall *p*^(1)^		0.007	0.006	0.010
	Pairwise *p*^(2)^ (evening vs. morning)		0.002	0.002	0.005
	Pairwise *p*^(2)^ (intermediate vs. morning)		0.642	0.758	0.842
	Adjusted *R*^2^^(3)^		0.115	0.099	0.120
Lactate	Morning	55	1.878 (1.694–2.061)	1.877 (1.671–2.083)	1.970 (1.746–2.195)
	Intermediate	61	1.984 (1.792–2.175)	2.015 (1.782–2.247)	2.058 (1.810–2.305)
	Evening	11	2.443 (2.052–2.835)	2.462 (2.048–2.875)	2.455 (2.037–2.873)
	Overall *p*^(1)^		0.032	0.032	0.097
	Pairwise *p*^(2)^ (evening vs. morning)		0.009	0.009	0.031
	Pairwise *p*^(2)^ (intermediate vs. morning)		0.393	0.288	0.506
	Adjusted *R*^2^^(3)^		0.046	0.010	0.050
Citrate	Morning	54	0.063 (0.060–0.066)	0.064 (0.061–0.067)	0.064 (0.061–0.068)
	Intermediate	61	0.060 (0.057–0.062)	0.062 (0.058–0.065)	0.061 (0.057–0.065)
	Evening	10	0.056 (0.049–0.062)	0.058 (0.052–0.065)	0.058 (0.051–0.064)
	Overall *p*^(1)^		0.043	0.158	0.102
	Pairwise *p*^(2)^ (evening vs. morning)		0.029	0.089	0.066
	Pairwise *p*^(2)^ (intermediate vs. morning)		0.059	0.161	0.095
	Adjusted *R*^2^^(3)^		0.103	0.139	0.167

Data are presented as least-square means (95% confidence intervals). The unit of measurement is mmol/L.

(1) *p*-values for the overall effect of chronotype were derived from general linear models with hierarchical adjustment for covariates in each model.

(2) Pairwise *p*-values for the evening versus morning and intermediate versus morning comparisons were obtained using Tukey–Kramer post-hoc tests.

(3) Adjusted *R*^2^ values indicate the proportion of variance explained by each model after accounting for the number of covariates.

(4) Metabolic disease-related medications included those for diabetes, hypertension, dyslipidemia, and ischemic heart disease.

Model 1: Adjusted for age, sex, and fasting status. Model 2: Adjusted for Model 1 covariates plus education level and household income. Model 3: Adjusted for Model 2 covariates plus alcohol consumption, smoking status, body mass index, and total energy intake.

## 4 Discussion

Pyruvate concentrations were consistently higher in the evening chronotype than in the morning chronotype across all models, and both overall and pairwise associations remained statistically significant after adjusting for potential confounders. Lactate concentrations also tended to be higher in the evening chronotype, with significant differences between the evening and morning chronotypes observed across all models. However, the overall effect of chronotype was no longer significant after covariate adjustment. Citrate concentrations showed the opposite pattern, decreasing from the morning to the evening chronotype. Although the overall effect of chronotype did not reach statistical significance, pairwise comparisons demonstrated lower citrate concentrations in the evening chronotype than in the morning chronotype in several models. Collectively, these findings suggest that the association between chronotype and pyruvate was the most robust, whereas associations with lactate and citrate were more sensitive to covariate adjustment, potentially reflecting the influence of multiple behavioral and metabolic factors on these metabolites. In addition, the pyruvate/lactate ratio, a proposed marker of mitochondrial redox state, was not significantly associated with chronotype.

Although the direction of the association for lactate was generally consistent with experimental evidence, the attenuation of the overall association after comprehensive adjustment suggests that lactate may be more susceptible to behavioral and metabolic influences than pyruvate. Similarly, citrate concentrations showed a consistent decreasing trend from the morning to the evening chronotype; however, the overall associations did not remain statistically significant after adjustment. These findings suggest that associations involving lactate and citrate should be interpreted more cautiously than those involving pyruvate. Moreover, the absence of a significant association for the pyruvate/lactate ratio indicates that the observed differences in pyruvate concentrations should not be interpreted as direct evidence of altered mitochondrial function.

From a metabolic perspective, pyruvate occupies a central position, linking glycolysis to mitochondrial oxidative metabolism (14), which may contribute to the stability of its association across models. In contrast, circulating lactate and citrate concentrations reflect the integration of multiple metabolic processes, including lactate recycling and alternative metabolic pathways (25). This complexity may reduce the ability to detect differences in circulating concentrations, particularly in cross-sectional studies in which dynamic metabolic flux through mitochondrial oxidative pathways or the TCA cycle cannot be directly assessed (26, 27).

Taken together, although glycolysis- and TCA-related metabolites may be associated with chronotype-related metabolic variation, the present findings suggest that these associations are modest and less consistent than those observed for pyruvate. Further longitudinal and mechanistic studies are needed to clarify the temporal and functional significance of these relationships.

Given the substantial imbalance in sample size across chronotype categories, particularly the relatively small number of participants classified as having an evening chronotype, chronotype was also examined as a continuous MEQ-reduced score. In the categorical analyses, participants with an evening chronotype exhibited significantly higher serum pyruvate concentrations, whereas lactate and citrate demonstrated statistically significant differences only in selected models. In contrast, when chronotype was modeled as a continuous MEQ-reduced score, the associations with pyruvate and lactate were attenuated after adjustment for covariates, whereas citrate showed a significant positive association with the MEQ-reduced score, indicating higher citrate concentrations with increasing MEQ-reduced scores (i.e., toward the morning chronotype; Supplementary Figure 1). This discrepancy may reflect differences in the analytical focus of the two approaches. Categorical models emphasize mean differences between predefined groups, whereas continuous analyses evaluate linear trends across the full distribution of MEQ-reduced scores (28).

These findings are broadly consistent with previous animal and experimental studies that reported altered mitochondrial oxidative metabolism and TCA cycle activity under conditions of circadian disruption (29, 30). Animal studies have shown that alterations in circadian clock gene expression and circadian misalignment are associated with reduced mitochondrial function and oxidative efficiency (31, 32). Although direct mechanistic inferences cannot be drawn from this cross-sectional study, the findings provide preliminary human evidence of chronotype-associated variation in circulating TCA-related metabolites.

Recent evidence suggests that nutrient-sensing pathways, particularly mechanistic target of rapamycin (mTOR) signaling, may represent an important molecular link between circadian regulation, mitochondrial metabolism, and metabolic homeostasis (33–36). Experimental and review studies have shown that mTOR interacts with circadian regulatory mechanisms to coordinate nutrient sensing, energy metabolism, mitochondrial function, and cellular adaptation to nutritional status (33–36). Dysregulation of these pathways has been implicated in altered metabolic flexibility and impaired metabolic homeostasis under conditions of circadian disruption (33–36). Although mTOR-related pathways were not directly evaluated in the present study, the observed chronotype-associated differences in circulating metabolites may be relevant to these emerging regulatory networks and warrant further investigation.

Several limitations should be acknowledged. First, the relatively modest sample size and recruitment from a single geographic region—a rural, mountainous area of Gangwon Province—may limit the generalizability of the findings to urban populations with different environmental exposures. Second, the imbalance in chronotype group sizes, particularly the small number of participants with an evening chronotype, necessitated the use of nonparametric statistical methods. This distribution may reflect the lifestyle characteristics of the community-dwelling population, including social norms favoring earlier wake times. Third, the cross-sectional design precludes causal inference because exposure and outcome were assessed at a single time point. Forth, only three metabolites were measured, limiting our ability to comprehensively assess mitochondrial function or TCA cycle activity. Finally, information on the exact time of blood collection was unavailable. Therefore, although fasting status was included as a covariate, the potential influence of circadian variation in metabolite concentrations cannot be completely excluded.

Future longitudinal studies are needed to determine whether chronotype-related differences in metabolite concentrations persist over time and to further investigate their biological significance. Additionally, intervention studies involving changes in sleep–wake schedules or meal timing across more diverse populations, including urban populations where eveningness is more prevalent, may provide stronger evidence of the causal relationship between social rhythms and mitochondrial metabolic profiles.

Nevertheless, these limitations may also be viewed as strengths. The relatively homogeneous nature of the cohort minimized variability in anthropometric characteristics (Supplementary Table 1), biochemical parameters (Supplementary Table 2), dietary intake (Supplementary Table 3), and NOVA-defined ultraprocessed food consumption (Supplementary Table 4), thereby enhancing internal validity by reducing potential confounding. In addition, the observed associations persisted after adjusting for fasting status, suggesting that the findings are unlikely to be explained solely by differences in pre-sampling fasting conditions. The absence of significant differences in sleep duration and social jetlag further supports the interpretation that the observed metabolite associations are not attributable solely to sleep-related factors. To our knowledge, this is the first human study to simultaneously evaluate serum concentrations of pyruvate, lactate, and citrate—key metabolites involved in glycolytic–mitochondrial integration—in relation to chronotypes. By focusing on metabolic intermediates, the findings provide a novel mechanistic perspective on how eveningness may be associated with variation in mitochondrial oxidative metabolism and TCA cycle-related pathways in a community-based setting.

In conclusion, our findings demonstrate differences in TCA cycle-related metabolites according to chronotypes under controlled fasting conditions. Specifically, individuals with an evening chronotype exhibited significantly higher serum pyruvate concentrations, and this association became more pronounced after excluding participants using metabolic disease-related medications. These results suggest that chronotype may be associated with variation in circulating pyruvate concentrations and related metabolic pathways. Future longitudinal and interventional studies are needed to clarify the underlying causal pathways and determine whether chronotype-informed approaches could improve early risk assessment and prevention of metabolic diseases.

## Data Availability

The dataset used and/or analyzed in this study is part of a limited cohort and is therefore not publicly available. The data may be obtained from the relevant authors or principal investigators upon reasonable request and appropriate permission.
