# Supplementary material for: Evening chronotype is associated with higher circulating pyruvate levels in adults
Source: Front Nutr. 2026 Jul 23;13:1839777. doi: 10.3389/fnut.2026.1839777 (PMC13442444; doi:10.3389/fnut.2026.1839777)
Supplement: Supplementary file 1 [file Data_Sheet_1.pdf]

Supplementary Material

Supplementary Methods. Blood biochemical parameters were analyzed using standard laboratory methods

**Analysis method**	**Blood biochemical parameters**
Enzymatic	Aspartate aminotransferase (AST), Alanine aminotransferase (ALT), Triglycerides (TGs), Total cholesterol
ECLIA	Insulin
UV assay (HK)	Glucose
Enzymatic assay	Low-Density Lipoprotein (LDL)-Cholesterol, High-Density Lipoprotein (HDL)-Cholesterol

Supplementary Table 1. Anthropometric characteristics and metabolic syndrome prevalence of study participants according to chronotype assessed using the Morningness–Eveningness Questionnaire-reduced

	Total (n = 272)			
	**Morning-chronotype (n = 132)**	**Intermediate-chronotype (n = 120)**	**Evening-chronotype (n = 20)**	***p-value***
**Anthropometrics**				
Body Mass Index (BMI, kg/m^2)	24.9 ± 0.4	25.4 ± 0.4	25.3 ± 0.9	0.646
Waist-to-hip ratio	0.90 ± 0.00	0.9 ± 0 .00	0.90 ± 0.01	0.305
Fat Mass (kg)	18.3 ± 0.2	18.8 ± 0.2	18.7 ± 0.5	0.152
Body fat percentage (%)	27.9 ± 0.3	28.5 ± 0.3	28.3 ± 0.7	0.308
Visceral fat area (cm^2)	103.3 ± 2.5	104.6 ± 2.8	104.6 ± 6.1	0.916
Visceral fat mass (kg)	2.4 ± 0.1	2.5 ± 0.1	2.5 ± 0.1	0.476
Systolic blood pressure	130.4 ± 1.6	128.6 ± 1.8	126.9 ± 3.8	0.564
Diastolic blood pressure (mmHg)	80.7 ± 1.0	78.2 ± 1.1	76.3 ± 2.3	0.071
**MetS[2)]**	32(24.2)	22(18.3)	5(25.0)	0.481

Data are presented as mean ± standard error (SE) or frequency (%).

p-Values for categorical variables were obtained using Fisher's exact test.

p-Values for continuous variables were derived from general linear models adjusted for age, sex, fasting status, education level, household income, body mass index (BMI), alcohol consumption, and smoking status.

[(1)] MetS = metabolic syndrome.

MetS was defined as meeting at least two of the following four criteria of the ATP III:

- Triglycerides ≥ 150 mg/dL or treatment for elevated triglycerides
- High-density lipoprotein cholesterol < 40 mg/dL in men or < 50 mg/dL in women
- Blood pressure ≥ 130/85 mmHg or antihypertensive treatment
- Fasting glucose ≥ 100 mg/dL or treatment for diabetes

Supplementary Table 2. Blood parameters of study participants according to chronotype assessed using the Morningness–Eveningness Questionnaire–reduced

	Total (n = 272)			
	**Morning-chronotype (n = 132)**	**Intermediate-chronotype (n = 120)**	**Evening-chronotype (n = 20)**	***p*-value**
**Blood parameters**				
Aspartate aminotransferase (IU/L)	28.3 ± 0.9	28.0 ± 1.1	23.3 ± 2.3	0.109
Alanine aminotransferase (IU/L)	25.8 ± 1.3	26.4 ± 1.4	21.4 ± 3.1	0.292
Insulin (*n*g/mL)	8.4 ± 0.7	9.6 ± 0.8	9.4 ± 1.7	0.423
Glucose (mg/dL)	107.2 ± 2.4	108.7 ± 2.7	102.6 ± 5.9	0.602
Triglycerides (mg/dL)	119.7 ± 7.0	113.7 ± 7.9	135.3 ± 17.1	0.461
Total cholesterol (mg/dL)	185.2 ± 4.0	179.7 ± 4.5	191.1 ± 9.8	0.413
LDL-cholesterol (mg/dL)	106.3 ± 3.5	99.2 ± 4.0	108.1 ± 8.5	0.269
HDL-cholesterol (mg/dL)	53.6 ± 1.3	55.0 ± 1.5	55.0 ± 3.1	0.718

Data are presented as mean ± standard error (SE).

p-Values were derived from general linear models adjusted for age, sex, fasting status, education level, household income, body mass index (BMI), alcohol consumption, and smoking status.

Supplementary Table 3. Comparison of nutrient intake assessed by a food frequency questionnaire according to chronotype assessed using the Morningness–Eveningness Questionnaire-reduced

	Total (n = 272)			
	**Morning-chronotype (n = 132)**	**Intermediate-chronotype (n = 120)**	**Evening-chronotype (n = 20)**	***p*-value**
**Nutrition intakes**				
Total Energy Intake (kcal)	2040.7 ± 77.5	2045.8 ± 83.8	1988.2 ± 179.0	0.953
Carbohydrates (E%)	61.5 ± 1.0	58.2 ± 1.2	59.0 ± 2.6	0.069
Protein (E%)	14.0 ± 0.3	14.6 ± 0.4	14.2 ± 0.8	0.370
Fat (E%)	18.8 ± 0.6	20.7 ± 0.7	18.5 ± 1.6	0.083
Saturated Fat (%E)	5.4 ± 0.2	5.9 ± 0.2	5.6 ± 0.5	0.111
Monounsaturated Fatty Acid (E%)	5.8 ± 0.2	6.5 ± 0.3	6.0 ± 0.6	0.101
Polyunsaturated Fatty Acid (E%)	5.1 ± 0.2	5.5 ± 0.2	4.5 ± 0.5	0.093
Omega-3 Fatty Acid (E%)	0.6 ± 0.0	0.7 ± 0.0	0.5 ± 0.1	0.159
Omega-6 Fatty Acid (E%)	4.5 ± 0.2	5.0 ± 0.2	4.0 ± 0.5	0.087
Sodium (mg)	3241.0 ± 176.4	3604.9 ± 206.1	3141.1 ± 444.5	0.277
Sodium density/1,000 kcal (mg)	1652.7 ± 61.5	1795.1 ± 71.8	1601.7 ± 154.9	0.187
Potassium (mg)	2902.5 ± 133.0	2938.8 ± 155.4	2378.0 ± 335.1	0.275
Potassium density/1,000 kcal (mg)	1498.6 ± 49.3	1497.9 ± 57.6	1232.6 ± 124.1	0.109

Fiber (g)	22.9 ± 1.2	22.3 ± 1.3	16.9 ± 2.9	0.142
Fiber density/1,000 kcal (g)	11.7 ± 0.4[b]	11.4 ± 0.5[ab]	9.0 ± 1.0[a]	**0.041**
Total cholesterol (mg/day)	301.5 ± 19.5	309.1 ± 22.8	272.1 ± 49.1	0.769
Calcium (mg/day)	496.0 ± 24.0	484.1 ± 28.0	368.3 ± 60.4	0.189
Vitamin C (mg/day)	105.3 ± 7.5	100.7 ± 8.8	62.0 ± 19.0	0.095

Data are presented as mean ± standard error (SE).

p-Values were derived from general linear models adjusted for age, sex, fasting status, education level, household income, body mass index (BMI), alcohol consumption, and smoking status.

Supplementary Table 4. Comparison of NOVA-defined ultra-processed food (UPF) intake according to chronotype assessed using the Morningness–Eveningness Questionnaire-reduced

	Total (n = 272)			
	**Morning-chronotype (n = 132)**	**Intermediate-chronotype (n = 120)**	**Evening-chronotype (n = 20)**	***p-value***
**NOVA-defined UPF**				
NOVA1 (%kcal/day)	54.4 ± 1.6	50.9 ± 1.8	50.9 ± 3.9	0.255
NOVA2 (%kcal/day)	0.5 ± 0.2	0.4 ± 0.2	0.8 ± 0.4	0.532
NOVA3 (%kcal/day)	24.0 ± 1.0	24.8 ± 1.2	22.7 ± 2.6	0.690
NOVA4 (%kcal/day)	21.2 ± 1.3	24.0 ± 1.5	25.7 ± 3.3	0.197

Data are presented as mean ± standard error (SE).

p-Values were derived from general linear models adjusted for age, sex, fasting status, education level, household income, body mass index (BMI), alcohol consumption, and smoking status.

Supplementary Table 5. Adjusted associations of chronotype with serum pyruvate / lactate ratio in all participants and after excluding participants using metabolic disease–related medications

All participants (n = 272)

	Chronotypes	n	Model 1	Model 2	Model 3
**Pyruvate / Lactate ratio**	Morning	132	0.043 (0.033–0.053)	0.043 (0.032–0.053)	0.043 (0.033–0.053)
	Intermediate	120	0.043 (0.033–0.053)	0.042 (0.032–0.052)	0.043 (0.032–0.053)
	Evening	20	0.043 (0.033–0.054)	0.042 (0.031–0.053)	0.043 (0.032–0.054)
	**Overall p[1)]**		0.979	0.961	0.950
	**Pairwise p[2)] (Evening vs. Morning)**		0.982	0.920	0.892
	**Pairwise p[2)] (Intermediate vs. Morning)**		0.840	0.780	0.752
	**Adjusted R^2 [3)]**		0.026	0.016	0.034

Participants without metabolic disease–related medication[4)] use (n = 127)

	Chronotypes	n	Model 1	Model 2	Model 3
**Pyruvate / Lactate ratio**	Morning	55	0.028 (0.025–0.031)	0.028 (0.025–0.031)	0.028 (0.025–0.032)
	Intermediate	61	0.028 (0.025–0.031)	0.027 (0.023–0.031)	0.027 (0.023–0.031)
	Evening	11	0.029 (0.022–0.035)	0.029 (0.022–0.035)	0.029 (0.022–0.036)
	**Overall p[1)]**		0.959	0.816	0.753

**Pairwise *p*[2)] (Evening vs. Morning)**	0.812	0.862	0.879
**Pairwise *p*[2)] (Intermediate vs. Morning)**	0.938	0.602	0.521
**Adjusted R^2 [3)]**	-0.015	-0.036	-0.062

Data are presented as least-square means (95% confidence intervals).

[(1)] *p*-Values for the overall effect of chronotype were derived from general linear models with hierarchical adjustment for covariates in each model.

[(2)] Pairwise *p*-values for the Evening versus Morning and Intermediate versus Morning comparisons were obtained using Tukey–Kramer post hoc tests.

[(3)] Adjusted R^2 values indicate the proportion of variance explained by each model after accounting for the number of covariates.

[(4)] Metabolic disease–related medications included those for diabetes, hypertension, dyslipidemia, and ischemic heart disease.

Model 1: Adjusted for age, sex, and fasting status.

Model 2: Adjusted for Model 1 covariates plus education level and household income.

Model 3: Adjusted for Model 2 covariates plus alcohol consumption, smoking status, body mass index, and total energy intake.

Supplementary figrue 1. Partial correlations between Morningness–Eveningness Questionnaire(MEQ)-reduced scores and serum metabolites

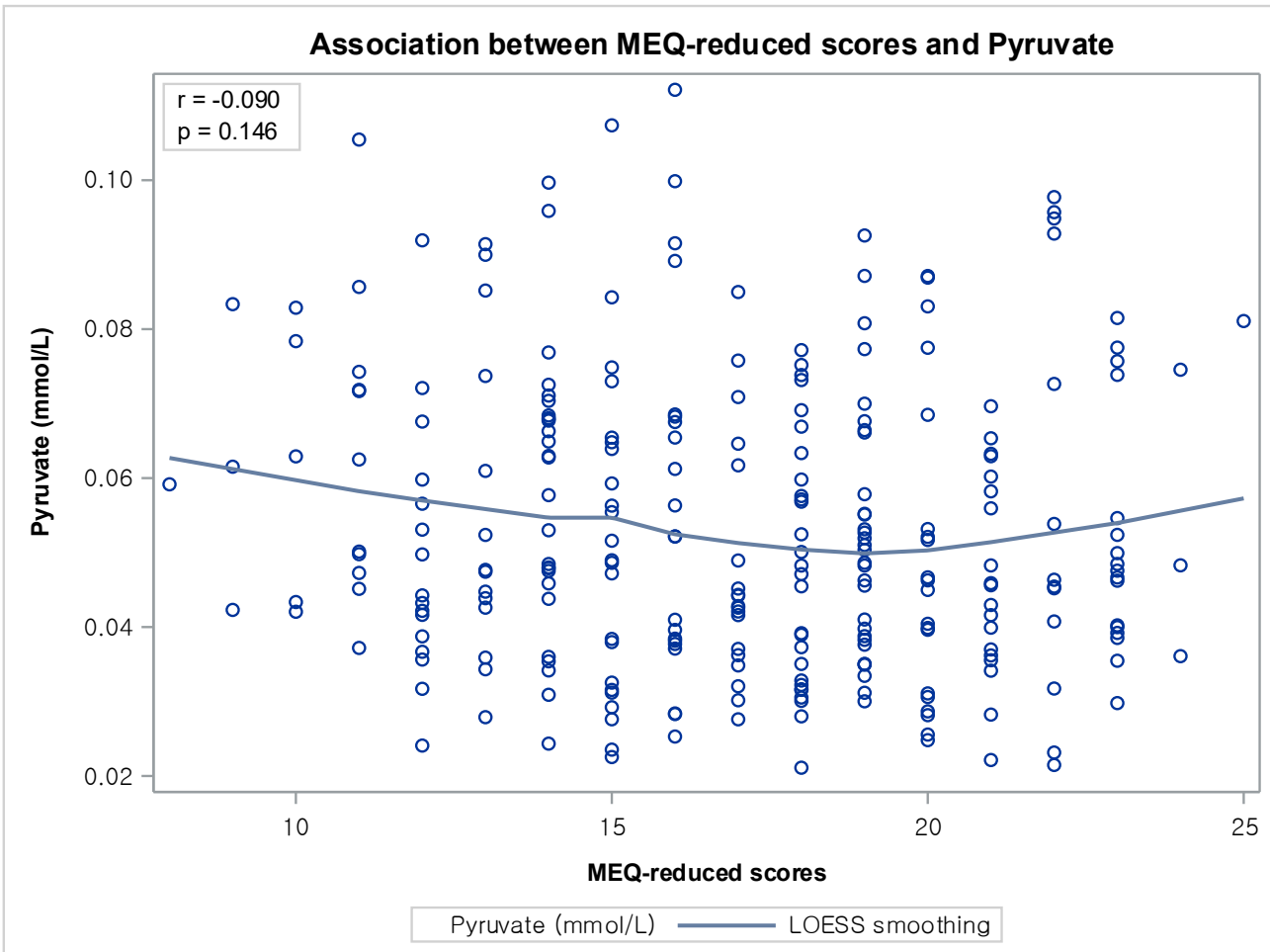

A. Serum pyruvate

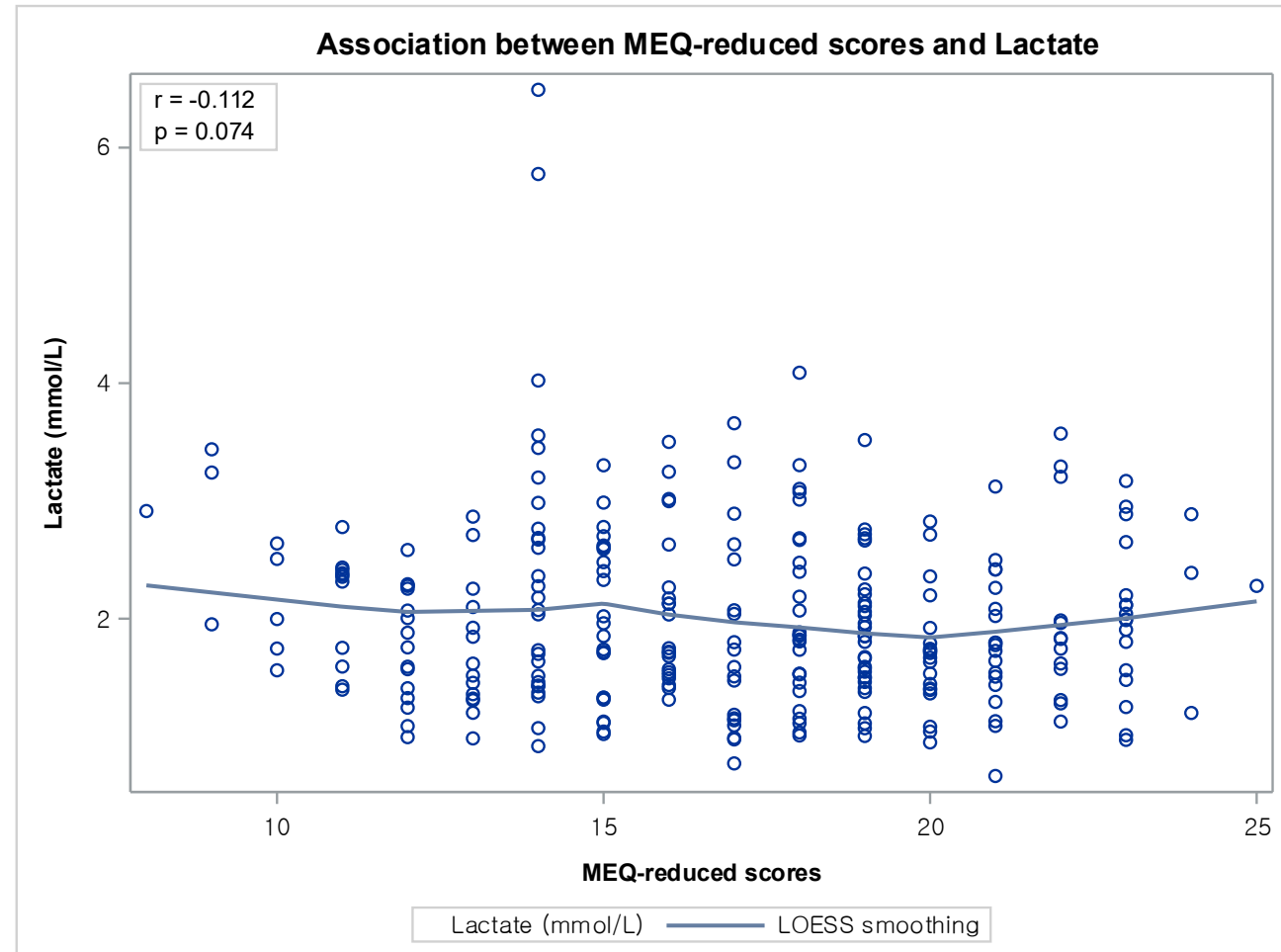

B. Serum lactate

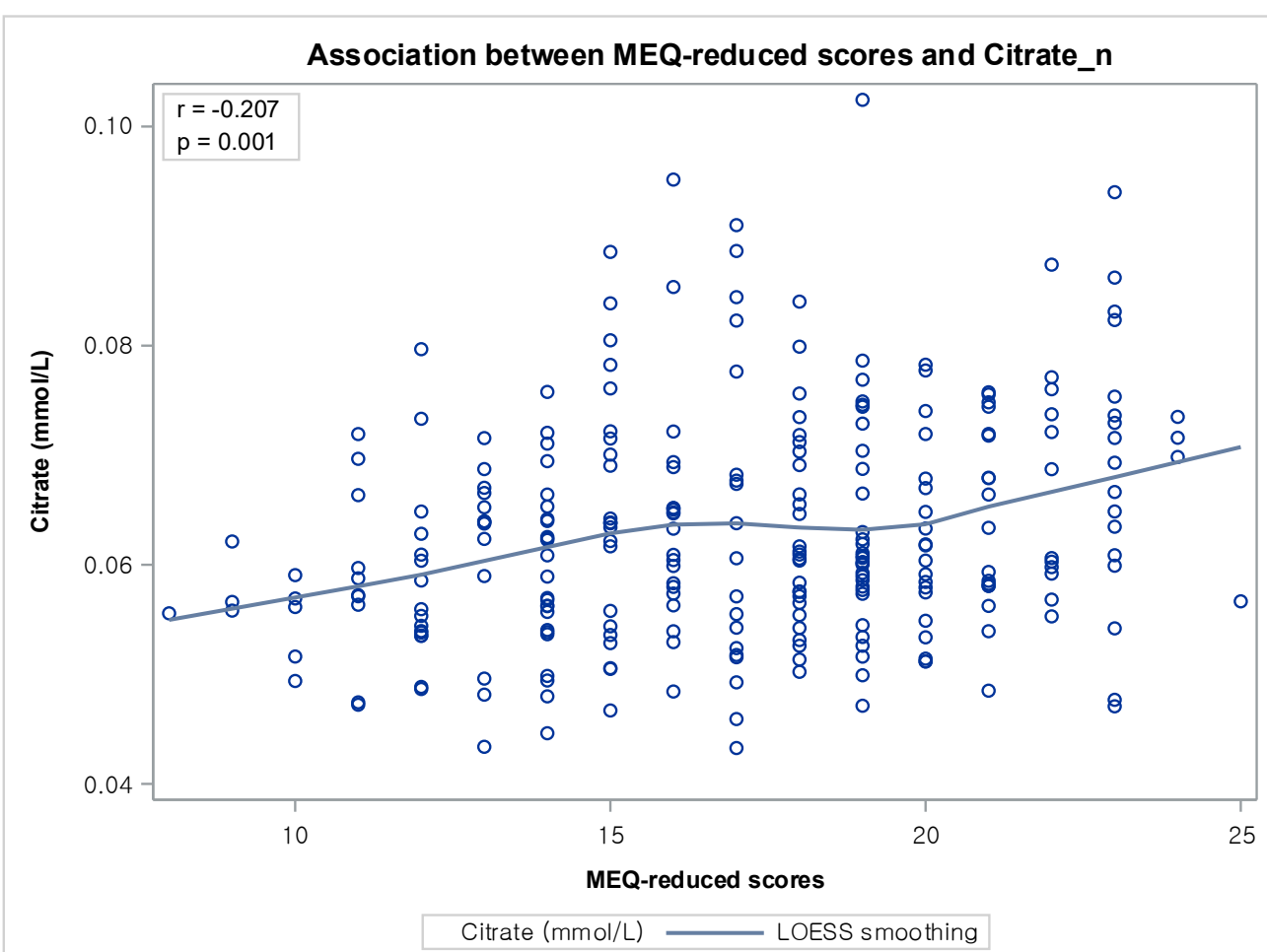

C. Serum citrate

p-values were obtained from partial correlation analyses adjusted for age, sex, fasting status, education, income, BMI, drinking, and smoking.
